# *In vitro* exposure to the agricultural triazole tebuconazole selects for fluconazole cross−resistance and echinocandin tolerance in *Candidozyma auris*

**DOI:** 10.3389/fcimb.2026.1860421

**Published:** 2026-07-20

**Authors:** Lihao Peng, Yi Xu, Xinyi Chen, Wenhui Li, Liangsheng Guo

**Affiliations:** 1 Jiading District Central Hospital, Affiliated to Shanghai University of Medicine and Health Sciences, Shanghai, China; 2 The 960th Hospital of PLA, Jinan, China; 3 Department of Obstetrics and Gynecology, The Second Affiliated Hospital of Soochow University, Suzhou, China

**Keywords:** Candida auris, echinocandins, medical azole, resistance, Tebuconazole, tolerance

## Abstract

The agricultural triazole fungicide tebuconazole (TCZ) has been implicated in the emergence of azole resistance in human fungal pathogens, but its direct role in selecting resistance and tolerance in *Candidozyma auris* remains unclear. Here, we investigated whether laboratory exposure of a clade III clinical *C. auris* isolate to TCZ could induce cross−resistance to clinical antifungals. Among 17 agrochemicals tested, only TCZ exhibited intrinsic antifungal activity. Brief (48 h) exposure to sub-MIC TCZ (1 μg/mL) selected variants (6.9% frequency) with cross-resistance to TCZ and fluconazole, but full susceptibility to caspofungin. Exposure to supra-MIC TCZ (8-32 μg/mL) produced three phenotypic classes: Class 2 (TCZ−R, fluconazole−R) and Class 3 (TCZ−R, fluconazole−R, plus tolerance to caspofungin and micafungin). The frequency of Class 3 increased dose-dependently from 23% to 50% as TCZ concentration rose. RNA-Seq revealed that both classes overexpressed *MDR1*, *TAC1b*, *UPC2* and *FKS1*, but Class 2 showed broad ergosterol pathway upregulation, whereas Class 3 exhibited restricted ergosterol activation but downregulation of the sole chitinase gene *CHT1* (5.17−fold), a known mechanism of echinocandin tolerance. Unlike the CRS−MIS phenomenon in *Candida glabrata*, our Class 3 adaptors displayed equal tolerance to both caspofungin and micafungin. Collectively, these findings demonstrate that, in a clade III clinical isolate of C. auris under the tested laboratory conditions, laboratory exposure to an agricultural triazole can rapidly select for clinically relevant azole cross−resistance and echinocandin tolerance in *C. auris*, suggesting that environmental fungicide use may inadvertently compromise the efficacy of last−line antifungals.

## Introduction

Since its first identification in 2009 from the ear canal of a patient in Japan (Satoh et al., 2009), *Candidozyma auris* (formerly *Candida auris*) has emerged as a formidable multidrug−resistant nosocomial fungal pathogen that poses a serious threat to global public health. Over the past decade, this yeast has rapidly spread to more than 40 countries across all populated continents, causing healthcare−associated outbreaks with alarming persistence and transmissibility (Kappel et al., 2024). The World Health Organization (WHO) has classified *C. auris* as a critical priority fungal pathogen, reflecting the urgent need for effective control and treatment strategies (WHO, 2022). Central to this threat is the unprecedented level of antifungal resistance exhibited by *C. auris*. Surveillance data indicate that up to 90% of clinical isolates are resistant to fluconazole—the most widely used clinical azole (Rybak et al., 2020)—and resistance to amphotericin B and echinocandins is increasingly reported, particularly in high−endemic settings (Chowdhary et al., 2018; Tian et al., 2024). Approximately 40% of clinical isolates display a multidrug−resistant pattern, and the mortality rate associated with *C. auris* candidemia averages around 30% (Cortegiani et al., 2019). Alarmingly, resistance is not intrinsic but acquired, with accumulating evidence showing that antifungal therapy can drive the stepwise development of multidrug resistance, including resistance to the last−line echinocandins through *FKS1* mutations (Carolus et al., 2021; Tian et al., 2024).

While *C. auris* is well recognized as a healthcare−associated pathogen, accumulating evidence has revealed that this yeast is not confined to clinical settings but also exists in the natural environment. For many years, the environmental reservoir of *C. auris* remained elusive. However, recent investigations have successfully isolated *C. auris* from diverse environmental niches. In 2021, viable *C. auris* was isolated from coastal wetlands of the Andaman Islands in India, including salt marshes, sand, and seawater, suggesting an association with marine ecosystems (Arora et al., 2021). More recently, a systematic review identified *C. auris* in a variety of environmental settings, including wastewater treatment plants, salt marshes, estuaries, dogs, and notably, the surfaces of apples (Silva et al., 2024). These discoveries collectively indicate that *C. auris* is not merely a transient human pathogen but possesses the capacity to persist and survive in environmental niches, raising important questions about the ecological drivers that may have shaped its extraordinary antifungal resistance profile.

A growing body of evidence suggests that the widespread use of azole fungicides in agriculture may serve as an environmental selective force driving the emergence of azole−resistant fungal pathogens—a hypothesis that has gained considerable traction in the context of the One Health framework (European Food Safety, A et al., 2025). This hypothesis was initially established for Aspergillus fumigatus, a ubiquitous environmental mold that causes life−threatening invasive aspergillosis in immunocompromised patients. Epidemiological and experimental studies have demonstrated that exposure to agricultural azole fungicides, particularly tebuconazole (TCZ) and other triazole−based plant protection products, can select for cross−resistance to clinical azoles in *A. fumigatus* (Alvarez-Moreno et al., 2019; Kang et al., 2022; Shelton et al., 2023; Meireles et al., 2025). A multi−agency report by the European Food Safety Authority and the European Centre for Disease Prevention and Control concluded that azole usage outside the human domain is likely or very likely to contribute to the selection of azole−resistant *A. fumigatus* isolates that can cause severe disease in humans (European Food Safety, A et al., 2025). The same selective pressure has also been documented in environmental yeasts of clinical importance. In *Cryptococcus neoformans* and *Cryptococcus gattii*, exposure to TCZ induced *in vitro* cross−resistance between the agrochemical and clinical azoles (fluconazole, itraconazole, and ravuconazole) (Bastos et al., 2018). More recent studies have further shown that environmentally relevant concentrations of TCZ and other agricultural triazoles (difenoconazole, propiconazole, uniconazole) can drive the emergence of triazole−resistant *C. neoformans* in soil through upregulation of ERG11 and efflux pump genes (Peng et al., 2025a, Peng et al., 2025b; Yu et al., 2025; Zhang et al., 2025). These findings collectively support the paradigm that agricultural fungicide use can inadvertently promote the development of antifungal resistance in human fungal pathogens present in the environment.

While the agricultural–clinical cross−resistance paradigm is well established in *A. fumigatus* and *Cryptococcus* species, several unique aspects of *C. auris* biology render this connection particularly uncertain and worthy of investigation. First, unlike the soil−borne *A. fumigatus*, *C. auris* exhibits a distinctive global clonal structure with limited genetic diversity, and its recent isolation from postharvest fruit surfaces—rather than soil—suggests that exposure routes and selective pressures may differ substantially, potentially involving fungicide residues on stored produce (Yadav et al., 2022). Second, although *FKS1* hotspot mutations are known to confer echinocandin resistance, the molecular basis of echinocandin tolerance (i.e., survival without altered MIC) in *C. auris* remains poorly understood, particularly whether agricultural azole exposure can drive this phenotype through cell wall remodeling pathways independent of *FKS* mutations. Third, a distinctive tolerance phenotype—equal survival against both caspofungin and micafungin—has been noted in *C. auris*, contrasting with the well−characterized CRS−MIS (caspofungin−reduced susceptibility, micafungin−increased susceptibility) phenomenon in *C. glabrata*, and suggesting that *C. auris* may employ distinct, species−specific mechanisms (Healey et al., 2011). Therefore, while the One Health framework provides a compelling rationale, direct experimental evidence linking agricultural triazole exposure to clinically relevant antifungal phenotypes in *C. auris*—particularly echinocandin tolerance—has been lacking.

Notwithstanding these species−specific peculiarities, indirect evidence supporting this hypothesis has emerged from environmental surveillance. In 2022, several *C. auris* strains were isolated from the surfaces of stored apples in India. Intriguingly, *C. auris* was not recovered from freshly picked apples, suggesting that the contamination occurs during postharvest storage rather than in the orchard. Stored fruits are often treated with a broad range of fungicides, including demethylation inhibitors (DMIs), to prevent postharvest spoilage, and residues of these agricultural azoles were indeed detected on the apples. Whole−genome sequencing revealed that the *C. auris* isolates from apples were genetically diverse and exhibited broad phylogenetic distribution within clade I, and importantly, most of these isolates showed cross−resistance to both agricultural and clinical azoles (Yadav et al., 2022). These observations suggest that fungicide−treated stored fruits may serve as a significant environmental niche for the selection and transmission of azole−resistant *C. auris* strains.

To directly test whether TCZ exposure can indeed select for cross−resistance to fluconazole and tolerance to echinocandins in *C. auris*, we exposed a clinical isolate (clade III, B12037) to sub− and supra−inhibitory concentrations of TCZ and evaluated the emergence of these phenotypes. We further employed transcriptomic profiling to uncover the molecular underpinnings of the observed adaptive responses. Our findings provide direct experimental evidence that, in a clade III isolate, agricultural fungicide use may inadvertently compromise the efficacy of last−line clinical antifungals, underscoring the urgent need for integrated One Health surveillance spanning agricultural and clinical sectors. Further studies across additional clades and conditions are needed to determine the generalizability of these observations.

## Materials and methods

### Strains and growth conditions

The wild−type strain used in this study was the *C. auris* clade III isolate B12037. This strain was originally isolated from ear fluid of a patient in Canada in 2012 (Chow et al., 2018). No prior antifungal exposure history has been documented for this isolate. Stock cultures were maintained in 25% glycerol at –80 °C. For routine cultivation, cells were grown in yeast extract–peptone–dextrose (YPD) medium containing 1% (w/v) yeast extract, 2% (w/v) peptone, and 2% (w/v) D−glucose, at 30 °C with shaking at 150–200 rpm. When solid media were required, YPD was supplemented with 2% (w/v) agar. Drug solutions were prepared in dimethyl sulfoxide (DMSO) and stored at -20˚C.

### Broth microdilution

The assay was performed according to the Clinical and Laboratory Standards Institute (CLSI) guidelines (CLSI, 2017) with minor modifications. Briefly, yeast cells were harvested during logarithmic growth, washed twice with sterile distilled water, and then re-suspended in distilled water. The cell density was adjusted to 2.5 × 10^3^ cells/mL in YPD broth containing TCZ at concentrations ranging from 0.25 to 128 μg/mL. Aliquots of 200 μL of the cell suspension were dispensed into each well of 96−well microtiter plates. Plates were incubated statically at 30 °C for 48 h. Following incubation, cell growth was quantified by measuring the optical density at 600 nm (OD_600_) using a microplate reader. Each condition was tested in triplicate, and control wells containing YPD broth without TCZ were included to correct for background growth.

### Spot assay

Spot assays were performed as described by Li et al (Li et al., 2023). Briefly, cells were resuspended in distilled water at a density of 1 × 10^7^ cells/mL. Three-microliter aliquots of ten-fold serial dilutions were then spotted onto YPD agar plates containing the indicated drugs. The plates were incubated at 30 °C for 2 days, after which photographs were taken.

### Sub-MIC TCZ exposure

Approximately 2.5 × 10^3^ cells/mL of strain B12037 were inoculated into 1.5 mL of YPD broth containing 1 μg/mL TCZ. Three independent biological replicates were performed. After 48 h of incubation at 30 °C with shaking, the cultures were washed and diluted with distilled water. Approximately 300 cells from each culture were then spread onto YPD agar plates and incubated at 30 °C for 48 h. From each replicate culture, 107 colonies were randomly selected and assessed for TCZ resistance using a spot assay.

### Supra-MIC TCZ exposure

Cells of B12037 were resuspended in distilled water to a density of 1 × 10^7^ cells/mL. Aliquots of 100 μL of the cell suspension were spread onto YPD plates supplemented with TCZ at concentrations of 0, 8, 16, and 32 μg/mL. The plates were incubated at 30 °C for 5 days. Colonies that grew under these conditions were randomly selected, streaked onto YPD plates, and incubated at 30 °C for 36 h. For each adaptor, several colonies of similar size were picked and stored at –80 °C.

### Disk diffusion assay

Disk diffusion assays were conducted following the principles of the CLSI M44-A2 standard (CLSI, 2009) but were adapted for comparative phenotypic screening. Instead of Mueller-Hinton agar, we used YPD agar to ensure optimal and consistent growth of *C. auris*. Filter disks (6 mm diameter, 0.7 mm thickness) were impregnated with 5 µL of drug solutions to achieve the following loads: fluconazole (200 µg), caspofungin (50 µg), or micafungin (50 µg). These concentrations were empirically chosen to be higher than CLSI recommendations to ensure clearly defined zones of inhibition for the parental *C. auris* strain B12037, enabling robust detection of relative phenotypic shifts in the derived variants.

For the assay, strains were grown overnight, adjusted to an optical density corresponding to ~1 × 10^6^ cells/mL, and 100 µL was spread evenly onto YPD agar plates. The drug-impregnated disks were then placed at the center of each plate. Plates were incubated at 30 °C for 48 hours, after which high-resolution photographs were taken.

All zone measurements were performed quantitatively and objectively using the diskImageR package (Gerstein et al., 2016; Rosenberg et al., 2018). Photographs were processed through the diskImageR pipeline, which uses ImageJ to analyze pixel density and fits a logistic model to the data. This method measures the radius of inhibition (RAD) to quantify resistance and the fraction of growth (FoG) within the zone of inhibition to quantify tolerance. The RAD_20_ value (the radius at which growth is inhibited by 20% relative to maximum growth) was used as the primary measure of resistance. The FoG_20_ value (the area under the growth curve above the RAD_20_ point) was used as the primary measure of tolerance. All assays were performed in at least two independent biological replicates.

### Operational definitions of resistance and tolerance

Following the *diskImageR* framework (Gerstein et al., 2016), we defined resistance as a significant reduction in the radius of inhibition at 20% growth reduction (RAD_20_) relative to the parental strain. Tolerance was defined as a fraction of growth (FoG_20_) within the zone of inhibition, indicating the presence of a subpopulation capable of growing above the resistance point. FoG_20_ was calculated as the area under the pixel−density curve within the ZOI up to the RAD_20_ threshold, divided by the maximum growth area. A significant increase of FoG_20_ value was scored as tolerance only when observed consistently across at least two independent biological replicates. These criteria were applied *a priori* to classify adaptors into phenotypic classes.

### RNA-seq

One representative isolate from Class 2 and one representative isolate from Class 3 were selected for RNA−Seq analysis. For each of these two isolates, as well as for the parental strain B12037, three independent biological replicates were prepared. Test strains were grown in YPD broth from an initial OD_600_ of 0.1 until they reached the logarithmic phase (OD_600_ = 1.0). Cell pellets were collected by centrifugation at highest speed for 1 min. Total RNA extraction, purification, library construction, and sequencing were performed as described by Sun et al (Sun et al., 2023). Briefly, total RNA was extracted using YeaStar™ RNA Kit (Zymo Research). The concentration and integrity of RNA in each sample were assessed by a Qubit RNA Assay Kit on a Qubit 2.0 Fluorometer (Life Technologies, CA, USA) and RNA Nano 6000 Assay Kit with the Bioanalyzer 2100 system (Agilent Technologies, CA, USA), respectively. RNA purity was determined using a Nano Photometer spectrophotometer (IMPLEN, CA, USA). The transcriptome libraries were constructed using the VAHTS mRNA-seq v2 Library Prep Kit (Vazyme Biotech Co., Ltd, Nanjing, China) according to the manufacturer’s instructions. RNA-seq was performed by BGI Genomics Co., Ltd. (Beijing, China). The samples were clustered using VAHRS RNA adapters set1/set2 and sequenced on an Illumina platform. The raw sequence files (.fastq files) underwent a quality control analysis using the FastQC tool (https://www.bioinformatics.babraham.ac.uk/projects/fastqc/). The reads were mapped to the *C. auris* clade III reference genome (https://www.candidagenome.org/download/sequence/C_auris_B11221/current/), and the genome annotation file (https://www.candidagenome.org/download/gff/C_auris_B11221/).

Differential expression analysis was conducted using DESeq2 (Love et al., 2014) with default parameters. Genes were considered differentially expressed if they met the following criteria: false discovery rate (FDR)-adjusted p-value < 0.05 and an absolute fold change > 2 (i.e., >2-fold upregulation or <0.5−fold downregulation).

### RNA extraction, synthesis of complementary DNA and quantitative real-time PCR

To compare gene expression between the parental strain and the adapted variants, cells were cultured in YPD broth and harvested at the logarithmic phase (OD_600_ = 1.0). Total RNA was extracted using the YeaStar™ RNA Kit (Zymo Research) according to the manufacturer’s instructions. RNA concentration and purity were assessed using a NanoDrop 2000C spectrophotometer (Thermo Fisher Scientific) by measuring absorbance at 230 nm, 260 nm, and 280 nm. RNA integrity was additionally verified by electrophoresis on 1% agarose gels under both denaturing and non−denaturing conditions for selected samples.

RNA samples were treated with DNase I (Thermo Fisher Scientific) at 37 °C for 30 min following the manufacturer’s protocol. Approximately 1 µg of total RNA was reverse−transcribed using the High−Capacity cDNA Reverse Transcription Kit (Thermo Fisher Scientific).

Quantitative real−time PCR (qRT−PCR) was performed using the CFX96 Touch Real−Time PCR System (Bio−Rad) to quantify the expression of candidate genes. The housekeeping gene *ACT1* was used as the internal control. Relative gene expression was calculated using the 2−ΔΔCT method (Schmittgen and Livak, 2008). Each reaction was performed in triplicate, and mean relative expression values were determined for each gene. All primers used are listed in **Supplementary Table 1**.

### Measurement of cell wall chitin content

Cell wall chitin content was determined by quantifying glucosamine released from purified cell wall fractions following acid hydrolysis, based on previously established protocols (Yang et al., 2013). Briefly, test strains were grown in YPD broth from an initial OD_600_ of 0.1 until reaching 1.0. Cells were harvested, washed three times with sterile distilled water, and resuspended in the same buffer. Disruption was performed using 0.5−mm glass beads in a Mini−Beadbeater (BioSpec Products). The resulting cell debris was washed five times with 1 M NaCl, and cell wall material was extracted by boiling in SDS−MeOH extraction buffer (50 mM Tris, 2% SDS, 0.3 M β−mercaptoethanol, 1 mM EDTA, pH 8.0) at 100 °C for 10 min. The extracts were then washed three times with sterile distilled water and dried using a SpeedVac concentrator.

For chitin quantification, approximately 4 mg (dry weight) of the purified cell wall material was hydrolyzed in 1 mL of 6 M HCl at 100 °C for 17 h. After evaporating the acid at 65 °C, the hydrolysate was reconstituted in 1 mL of sterile distilled water. A 0.1−mL aliquot of the sample was mixed with 0.1 mL of 1.5 M Na_23_ containing 4% acetyl acetone and incubated at 100 °C for 20 min. Subsequently, 0.7 mL of 96% ethanol and 0.1 mL of Ehrlich’s reagent (1.6 g of p−dimethylaminobenzaldehyde dissolved in 30 mL of concentrated HCl and 30 mL of ethanol) were added. After a 1−h incubation at room temperature, the absorbance at 520 nm was recorded using a plate reader (SpectraMax M5; Molecular Devices). Glucosamine concentrations were determined by interpolation from a standard curve (0–0.5 mg/mL glucosamine; Sigma−Aldrich). Chitin content was finally expressed as a percentage of the cell wall dry weight.

### Statistical analysis

All experiments were performed in at least two independent biological replicates, and data are expressed as mean ± standard deviation (SD) unless otherwise indicated. Normality of data distribution was assessed using the Shapiro–Wilk test. For comparisons between two groups (e.g., parental strain vs adapted class), unpaired two-tailed Student’s t-tests were used for normally distributed data, and Mann–Whitney U tests were used for non−normally distributed data. For comparisons among three or more groups (e.g., Class 1 vs Class 2 vs Class 3), one−way ANOVA with Tukey’s post−hoc test was used for normally distributed data, and the Kruskal–Wallis test with Dunn’s post−hoc test was used for non−normally distributed data. For categorical data (e.g., class distribution across TCZ concentrations), chi−square tests were performed. All statistical analyses were conducted using GraphPad Prism 9 (GraphPad Software, San Diego, CA, USA) or R version 4.2.0 (R Foundation for Statistical Computing, Vienna, Austria). Exact p-values are reported in the Results section or figure legends, and p < 0.05 was considered statistically significant.

For the analysis of proportions, 95% confidence intervals were calculated using the Wilson score interval. For dose−dependent trends in categorical outcomes (e.g., Class 3 proportion across TCZ concentrations), the Cochran–Armitage test for trend was performed using the prop.trend.test() function in R. To estimate effect sizes, logistic regression was performed using the glm() function with family = binomial, with TCZ concentration treated as an ordinal predictor and Class 3 status (tolerant vs non−tolerant) as the binary outcome. Odds ratios and their 95% confidence intervals were calculated from the regression coefficients.

## Results

### Susceptibility of *C. auris* to agrochemicals

To assess whether agricultural fungicides could directly affect *C. auris*, we evaluated the antifungal activity of 17 agrochemicals representing diverse chemical classes against a clade III clinical isolate (B12037) of *C. auris*. The tested compounds included strobilurins (azoxystrobin, pyraclostrobin), a benzimidazole (benomyl), carboxamides (boscalid, flutolanil), a chloronitrile (chlorothalonil), an anilinopyrimidine (cyprodinil), an aromatic hydrocarbon (dichloran), a morpholine (dimethomorph), a benzamide (fluopicolide), a dicarboximide (iprodione), an organophosphate (iprobenfos), a dithiolane (isoprothiolane), a phenylamide (metalaxyl), a phthalimide (phthalide), a quinoline (pyroquilon), a triazole (tebuconazole), and a triazolobenzothiazole (tricyclazole). This selection encompassed multiple modes of action, including mitochondrial respiration inhibition (strobilurins, carboxamides), microtubule disruption (benzimidazole), sterol biosynthesis inhibition (triazoles), and cell wall/membrane targeting (morpholine, benzamide), thus providing broad coverage of potential antifungal mechanisms against this emerging pathogen. Detailed information on each agrochemical, including its chemical class and biological target, is provided in **Supplementary Table 1**.

**Figure 1:**
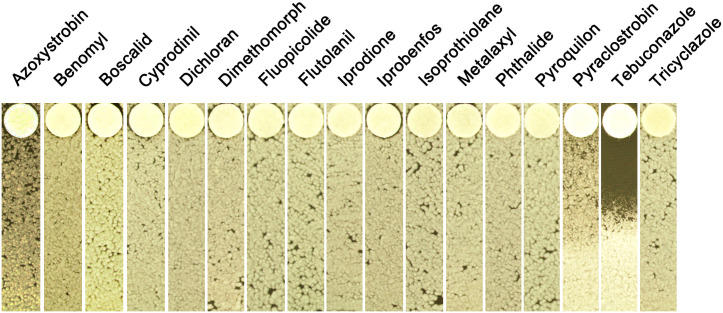
Disk diffusion assay of *C auris* against 17 agrochemicals. YPD-agar plates were inoculated with *C. auris* clade III strain B12037. 100 μg of each agrochemical was applied onto a sterile filter disk placed on the agar surfaceFor each compound, the disk is paced at the center of the plate. Some plates display a clear circular zone around the disk (TCZ), whereas other plates show slow fungal growth up to the disk edge within the zone (azoxystrobin and pyraclostrobin). The specific agrochemical tested is indicated on each plate.

We performed disk diffusion assays by applying 100 μg of each agrochemical onto YPD−agar plates inoculated with *C. auris* strain B12037. Among the 17 compounds tested, only TCZ produced a clear, well-defined inhibition zone, indicating significant antifungal activity against *C. auris* (**Figure 1**). In contrast, the remaining 16 agrochemicals either failed to inhibit fungal growth entirely or exhibited colonies growing within the inhibition zone (**Figure 1**). This latter phenotype—observed notably for the strobilurins azoxystrobin and pyraclostrobin—suggests that *C. auris* may display tolerance to these compounds under the tested conditions. Collectively, these results demonstrate that while most agricultural fungicides from diverse chemical classes lack intrinsic activity against *C. auris*, the triazole fungicide tebuconazole shows clear growth inhibition, prompting further investigation into its potential to select for cross−resistance to clinical azoles.

### Short−time exposure to sub−MIC concentration of TCZ selects adaptors cross−resistant to TCZ and fluconazole

**Figure 2:**
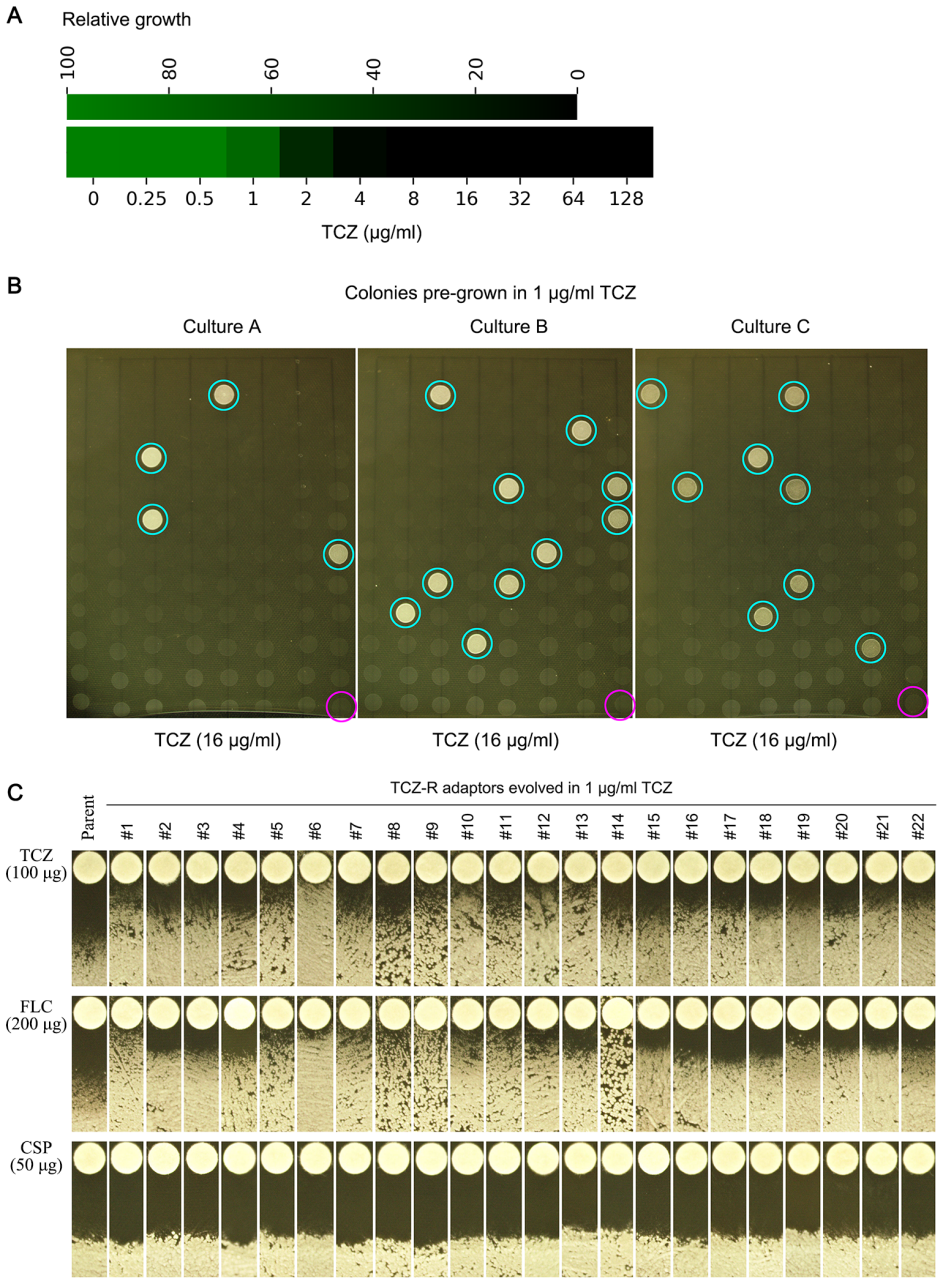
Selection of *C. auris* variants after short−term exposure to a sub−MIC concentration of TCZ. **(A)** Broth microdilution assay results for B12037 grown in the presence of increasing concentrations of TCZ. Shown is the percent growth in wells with drugs relative to the drug-free control. **(B)** Spot assay results of colonies derived from B12037 pre-exposed to 1 μg/mL TCZ. A total of 321 colonies (107 from each of three independent biological replicates) were spotted onto YPD-agar containing 2 μg/mL TCZ. Parental strain B12037 (without prior TCZ exposure) is included as a control. Cyan circles indicate colonies that grew robustly on the TCZ-containing plate; magenta circles indicate the parental strain spot. **(C)** Disk diffusion assays for the parental strain B12037 and 22 selected colonies (numbered or arranged in rows). Filter disks were impregnated with TCZ (100 μg), fluconazole (FLC, 200 μg), or caspofungin (CSP, 50 μg). Clear zones of growth inhibition (halos) are visible around each disk. For each condition, the plates are photographed after 48 h of incubation at 30 °C.

To determine whether even a brief exposure to low, sub−inhibitory concentrations of TCZ could induce resistance in *C. auris*, we first established the baseline susceptibility of the clade III strain B12037 to TCZ using the broth microdilution method. As shown in **Figure 2A**, growth of B12037 was clearly inhibited at 2 μg/mL TCZ, and this concentration was therefore defined as the minimum inhibitory concentration (MIC). To simulate a short-term environmental or agricultural exposure, cells of B12037 were pre-grown in liquid YPD medium containing 1 μg/mL TCZ (i.e., half the MIC) for 48 h. After this pre-exposure period, cells were harvested, washed, and plated onto YPD agar without any drug to allow colony formation.

From each of three independent biological replicate cultures, we randomly selected and picked 107 colonies (total of 321 colonies across replicates). These colonies were then screened for reduced susceptibility to TCZ using a spot assay on YPD agar containing 2 μg/mL TCZ. The parental strain B12037 (grown without prior TCZ exposure) was included as a control on each plate. Colonies that grew robustly under this condition were considered putatively resistant. In total, 22 colonies (indicated by cyan circles in **Figure 2B**) showed noticeably better growth on TCZ−containing medium compared to the parent strain (magenta circles in **Figure 2B**), representing an apparent adaptation frequency of approximately 6.9% (22/321) under the tested conditions.

To confirm the resistance phenotype and evaluate cross−resistance to clinically relevant antifungals, we performed disk diffusion assays for all 22 adapted colonies. Filter disks were impregnated with TCZ (100 µg), fluconazole (FLC, 200 µg), or caspofungin (CSP, 50 µg). After 48 h of incubation, inhibition zones were analyzed using *diskImageR* (Gerstein et al., 2016). The software quantifies resistance as the radius of inhibition at 20% growth reduction (RAD_20_) and tolerance as the fraction of growth within the zone of inhibition (FoG_20_).

Quantitative analysis using *diskImageR* revealed that, for the parental strain, the RAD_20_ values for TCZ, FLC, and CSP were 12.3 ± 0.58, 14.0 ± 0.00, and 12.7 ± 0.58, respectively. All 22 adapted colonies exhibited significantly reduced RAD_20_ values for TCZ (mean ± SD: 1.8 ± 1.6, range 0–3.5; p < 0.0001 vs parent, unpaired t−test) and FLC (mean ± SD: 1.1 ± 0.9, range 0–2.3; p < 0.0001 vs parent), whereas the RAD_20_ values for CSP remained unchanged (mean ± SD: 12.9 ± 0.2, range 12.7–13.0, p = 0.16 vs parent) (**Figure 2C**). FoG_20_ values for the parental strain were 0.15 ± 0.04, 0.25 ± 0.02, and 0.12 ± 0.01 for TCZ, FLC, and CSP, respectively, and no significant increase in FoG_20_ was observed for the adapted colonies compared to the parent for any of the three drugs (p > 0.05 for all, Mann–Whitney U tests).

Collectively, these results demonstrate that, compared with the parental strain, all 22 adapted colonies exhibited significantly reduced inhibition zones for both TCZ and FLC, indicating clear cross−resistance between the agricultural triazole and the clinical azole. In contrast, the inhibition zones for caspofungin remained essentially unchanged relative to the parent (**Figure 2C**). These findings demonstrate that short−term exposure to a sub−MIC concentration of TCZ is sufficient to select for *C. auris* variants that are cross−resistant to TCZ and FLC, while remaining fully susceptible to CSP.

### Exposure to supra-MIC concentration of TCZ selects adaptors cross−resistant to FLC and tolerant to CSP

**Figure 3:**
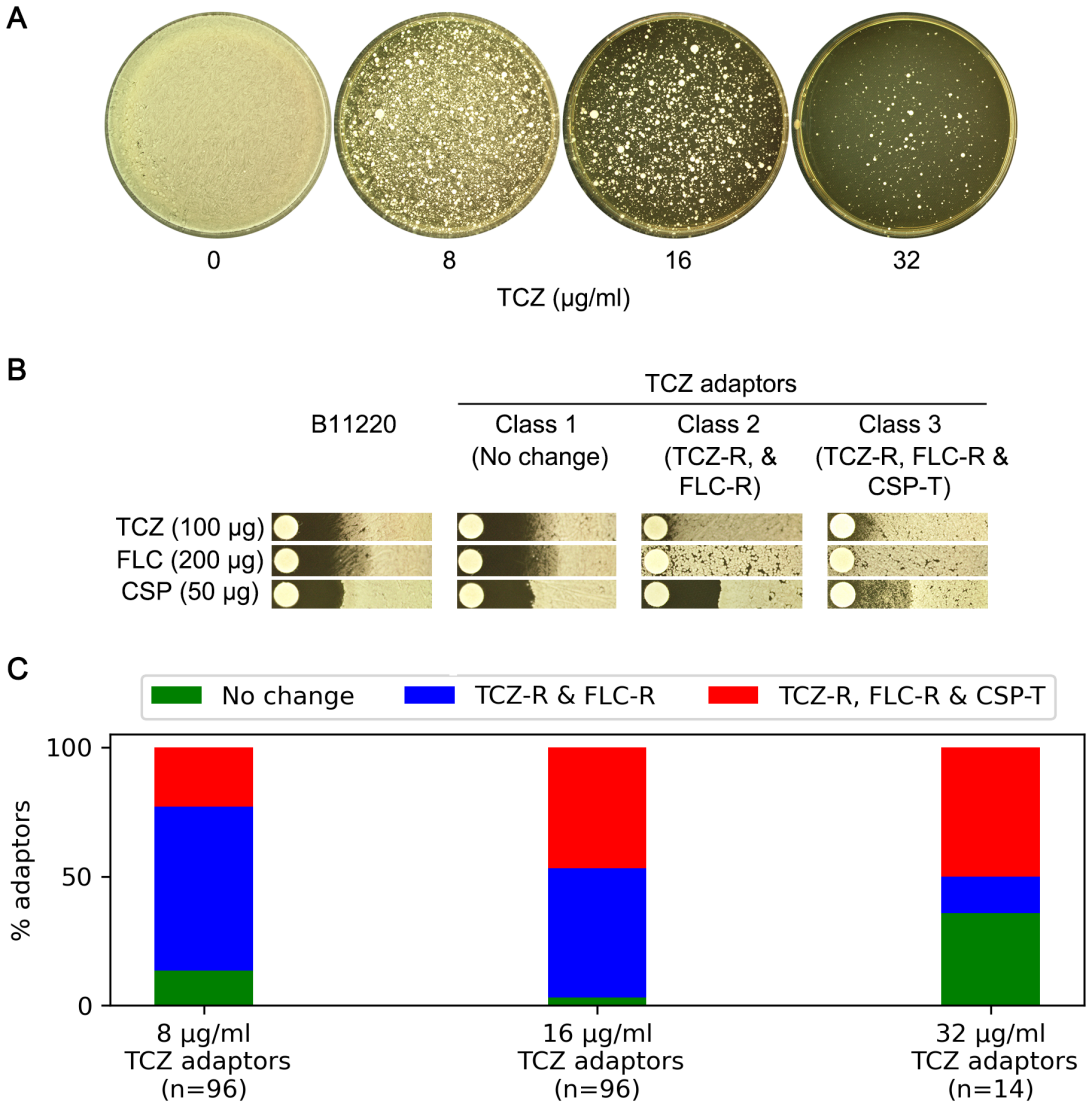
Phenotypic classification of *C. auris* adaptors selected on supra−MIC concentrations of TCZ. **(A)** Photographs of YPD-agar plates containing TCZ at 8 μg/mL, 16 μg/mL, and 32 μg/mL. Approximately 1 × 10^6^ cells of B12037 were spread onto each plate. Visible colonies are shown on the plates. **(B)** Disk diffusion assay results for three representative adaptor classes (Class 1, Class 2, and Class 3) compared to the parental strain. Filter disks are impregnated with TCZ (100 μg), fluconazole (FLC, 200 μg), or caspofungin (CSP, 50 μg). In some strains (e.g., Class 2 and Class 3 for TCZ and FLC), the inhibition zone diameter is reduced relative to the parent. For Class 3 with CSP, colonies are visible inside the inhibition zone, while the outer zone radius remains similar to that of the parent. **(C)** Stacked bar chart showing the distribution of the three classes among adaptors selected at 8 μg/mL, 16 μg/mL, and 32 μg/mL TCZ. The x-axis indicates the TCZ selection concentration. The y-axis indicates the percentage (%) of adaptors falling into each class.

To investigate the effect of a stronger selective pressure imposed by TCZ, we exposed B12037 to TCZ concentrations above the MIC. Approximately 1 × 10^6^ cells were spread onto YPD-agar plates containing TCZ at 8 μg/mL, 16 μg/mL, or 32 μg/mL (i.e., 4×, 8×, and 16× the MIC, respectively). After incubation, colonies that grew on these plates were selected as adaptors. From the 8 μg/mL plate, 96 colonies were picked; from the 16 μg/mL plate, another 96 colonies; and from the 32 μg/mL plate, 14 colonies (**Figure 3A**).

All selected adaptors were then subjected to disk diffusion assays to evaluate their susceptibility to TCZ, FLC and CSP. Interestingly, based on their response patterns, the adaptors could be divided into three distinct phenotypic classes based on the following explicit criteria (**Figure 3B**).

Class 1 adaptors showed no significant change in RAD_20_ (inhibition zone radius) for any of the three drugs compared to the parental strain, and no visible colonies within the zone of inhibition.

Class 2 adaptors (TCZ-R, FLC-R) exhibited a visibly reduced RAD_20_ for TCZ and FLC (cross−resistance), but no change in RAD_20_ for CSP and no colonies within the CSP zone of inhibition.

Class 3 adaptors (TCZ-R, FLC-R, CSP-T) exhibited visibly reduced RAD_20_ for TCZ and FLC (cross−resistance), plus visible colonies growing within the zone of inhibition for CSP (and MCF), while the outer RAD_20_ for CSP remained comparable to that of the parent—this latter feature defining tolerance.

Representative diskImageR analysis of each class confirmed the phenotypic classification. For Class 2 adaptors (n = 3 representative isolates), RAD_20_ values were significantly reduced for TCZ (mean ± SD: 2.1 ± 0.7; p < 0.001 vs parent) and FLC (mean ± SD: 1.3 ± 0.5; p < 0.001 vs parent), but remained unchanged for CSP (mean ± SD: 12.8 ± 0.4; p = 0.34 vs parent). For Class 3 adaptors (n = 3 representative isolates), RAD_20_ values were similarly reduced for TCZ (mean ± SD: 1.2 ± 0.6; p < 0.001 vs parent) and FLC (mean ± SD: 0.8 ± 0.7; p < 0.001 vs parent), while FoG_20_ values for CSP were significantly elevated compared to the parent (mean ± SD: 0.48 ± 0.06 vs 0.12 ± 0.01; p < 0.001, Mann–Whitney U test), confirming the tolerant phenotype.

The distribution of these three classes differed markedly depending on the TCZ concentration used for selection (**Figure 3C**). Among adaptors selected at 8 µg/mL TCZ, 63.5% fell into Class 2 and 23.0% into Class 3 (22/96; 95% CI: 15.6% – 32.3%), with the remainder (13.5%) showing no change (Class 1). At 16 µg/mL TCZ, the proportion of Class 2 adaptors decreased to 50%, while the proportion of Class 3 adaptors increased sharply to 46.9% (45/96; 95% CI: 37.1% – 56.9%). Among the few adaptors selected at 32 µg/mL TCZ, 50.0% (7/14; 95% CI: 26.8% – 73.2%) exhibited the Class 3 phenotype.

A Cochran–Armitage test for trend confirmed that the proportion of Class 3 adaptors increased significantly with increasing TCZ concentration (p = 0.002). Logistic regression further revealed that each doubling of the TCZ concentration (i.e., each one−step increase from 8 to 16 to 32 µg/mL) was associated with an odds ratio of 2.7 (95% CI: 1.5 – 5.2) for the emergence of the Class 3 (echinocandin−tolerant) phenotype.

These results demonstrate that higher TCZ selective pressure not only enriches for cross-resistance to the clinical azole FLC but also promotes the emergence of tolerance to the echinocandin CSP. Importantly, the frequency of CSP-tolerant adaptors increased in a dose-dependent manner with the TCZ concentration used during selection, suggesting that exposure to high levels of agricultural TCZ could drive the development of phenotypes that compromise the two main classes of clinical antifungals (azoles and echinocandins) used to treat *C. auris* infections.

### Characterization of echinocandin tolerance in TCZ-selected adaptors

**Figure 4:**
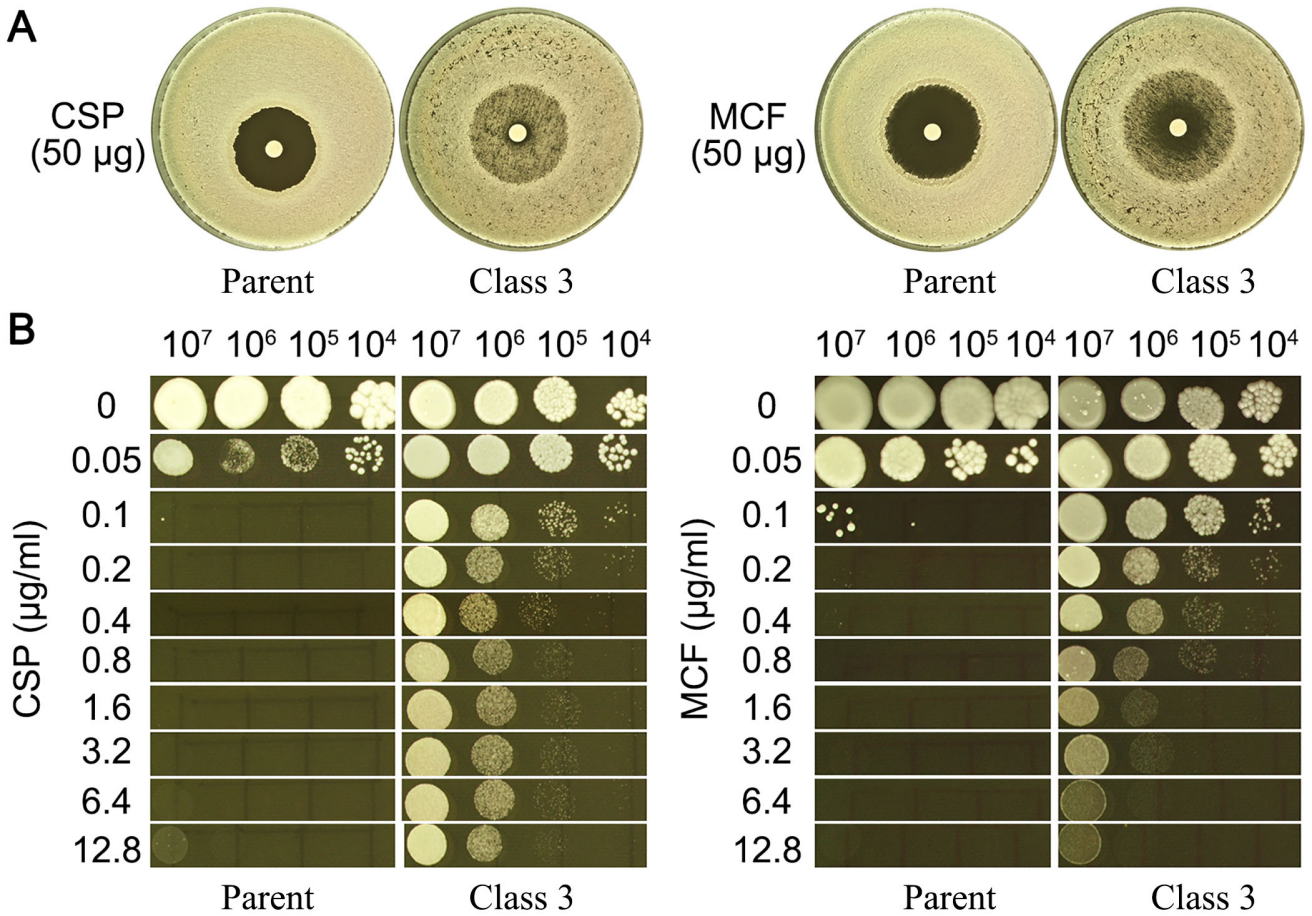
Susceptibility of Class 3 adaptor to caspofungin and micafungin. **(A)** Disk diffusion assay. Filter disks containing caspofungin (CSP, 50 μg) or micafungin (MCF, 50 μg) were placed on YPD−agar plates inoculated with either the parental C. auris strain B12037 (Parent) or a representative Class 3 adaptor. Clear circular inhibition zones (halos) are visible around each disk. For the parental strain, the inhibition zones are uniformly free of visible colonies. For the Class 3 adaptor, colonies are present within the inhibition zones around both the CSP and MCF disks, while the outer boundary of each zone remains visible. **(B)** Spot assay. Serial ten-fold dilutions of the parental strain and a Class 3 adaptor were spotted onto YPD-agar plates containing increasing concentrations of caspofungin (CSP, left panel) or micafungin (MCF, right panel), ranging from 0.05 to 12.8 μg/mL as indicated. Plates were photographed after 48h growth at 30 °C.

We next examined whether the Class 3 adaptors (TCZ-R, FLC-R, CSP-T) displayed altered susceptibility to other echinocandins. Disk diffusion assays and spot assays were performed using caspofungin (CSP, 50 μg) and micafungin (MCF, 50 μg). As shown in **Figure 4A**, Class 3 adaptor exhibited visible colonies growing inside the inhibition zones for both CSP and MCF, whereas the parental strain showed clear, colony-free zones around both disks. The outer radius of the inhibition zone remained unchanged for both drugs compared to the parent, confirming that the phenotype is tolerance (growth within the zone) rather than resistance (reduction of zone radius).

Quantitative *diskImageR* analysis further confirmed that Class 3 adaptors (n = 3 isolates) displayed significantly elevated FoG_20_ values for both CSP (mean ± SD: 0.45 ± 0.08; p < 0.001 vs parent, Mann–Whitney U test) and MCF (mean ± SD: 0.41 ± 0.07; p < 0.001 vs parent), while the outer RAD_20_ remained comparable to that of the parent (CSP: 12.5 ± 0.5 vs 12.7 ± 0.6, p = 0.42; MCF: 13.1 ± 0.4 vs 13.3 ± 0.5, p = 0.38).

Spot assays using a wide range of CSP and MCF concentrations (0.05-12.8 μg/mL) verified the ability of Class 3 adaptors to grow in the presence of both echinocandins across concentration range of 0.1-128 μg/mL. In contrast, the parental strain was completely inhibited by 0.1 μg/mL of either CSP or MCF (**Figure 4B**).

Notably, this pattern contrasts with a previously described phenomenon in Candida glabrata, where mutants selected for reduced caspofungin susceptibility paradoxically showed increased susceptibility to micafungin (a phenotype termed CRS−MIS) (Healey et al., 2011). In our *C. auris* adaptors, no such differential response was observed; instead, tolerance extended equally to both echinocandins, suggesting a distinct mechanism underlying echinocandin tolerance in this species.

### RNA-Seq reveals differential expression of drug resistance genes in TCZ−selected adaptors

To identify putative genes underlying the observed resistance and tolerance phenotypes, we performed transcriptome sequencing (RNA-Seq) on one representative Class 2 adaptor (cross-resistant to TCZ and FLC) and one representative Class 3 adaptor (cross-resistant to TCZ and FLC, plus echinocandins tolerance), comparing each to the parental strain B12037. Three biological replicates were prepared for each strain. Differential expression analysis was conducted using DESeq2 with default parameters. Genes were considered differentially expressed if they met the following criteria: false discovery rate (FDR)−adjusted p−value < 0.05 and an absolute fold change > 2 (i.e., > 2−fold upregulation or < 0.5−fold downregulation). Data are summarized as the mean expression values from the three replicates.

**Figure 5:**
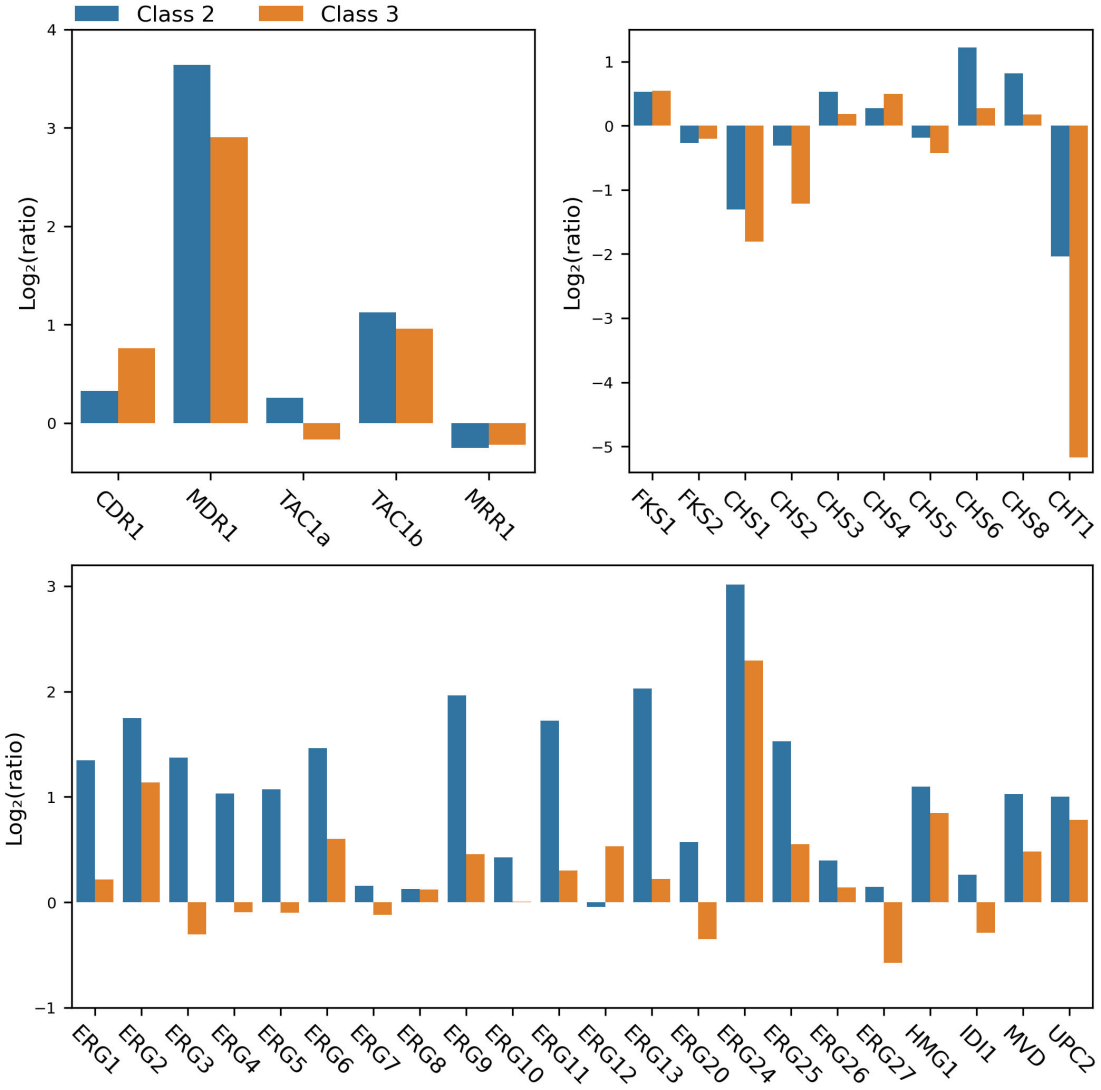
Bar chart showing expression levels of selected genes in the parental strain, Class 2 adaptor, and Class 3 adaptor. RNA-Seq was performed on the parental B12037, one representative Class 2 adaptor, and one representative Class 3 adaptor, with three biological replicates per strain. For each of the indicated genes, the y−axis represents the expression value (log_2_-transformed fold change relative to the parental strain). The x-axis lists the gene names. Genes are grouped into categories: drug efflux and transcription factors (*MDR1*, *CDR1*, *TAC1a*, *TAC1b*, *UPC2*); ergosterol biosynthesis pathway (*ERG1*, *ERG2*, *ERG3*, *ERG4*, *ERG5*, *ERG6*, *ERG9*, *ERG10*, *ERG11*, *ERG12*, *ERG13*, *ERG20*, *ERG24*, *ERG25*, *ERG26*, *HMG1*, *MVD*); and echinocandin target and cell wall genes (*FKS1*, *CHS1*, *CHS2*, *CHS3*, *CHS4*, *CHS6*, *CHS8*, *CSH1*, *CHT1*).

The multidrug resistance gene *MDR1* was overexpressed in both adaptors relative to the parent. The azole efflux pump gene *CDR1* was also overexpressed, but only in the Class 3 adaptor. Two variants of the transcription factor gene *TAC1* (a known regulator of drug efflux) are present in the *C. auris* genome: *TAC1a* (CJI97_004633) and *TAC1b* (CJI97_004632). Only *TAC1b* was overexpressed in both adaptors, whereas *TAC1a* showed no consistent change (**Figure 5**).

A marked difference was observed between the two adaptor classes in the expression of genes involved in ergosterol biosynthesis. In the Class 2 adaptor, a broad and coordinated upregulation of the ergosterol pathway was evident. Overexpressed genes included *ERG1*, *ERG2*, *ERG3*, *ERG4*, *ERG5*, *ERG6*, *ERG9*, *ERG10*, *ERG11*, *ERG13*, *ERG20*, *ERG24*, *ERG25*, *ERG26*, *HMG1*, and *MVD*. In contrast, the Class 3 adaptor exhibited a more restricted upregulation pattern, with only a subset of these genes showing increased expression: *ERG2*, *ERG6*, *ERG9*, *ERG12*, *ERG24*, *ERG25*, *HMG1*, and *MVD*. Importantly, the transcription factor *UPC2*, which is known to regulate ergosterol biosynthetic genes and sterol uptake, was overexpressed in both adaptors (**Figure 5**). This suggests that *UPC2* upregulation may be a common upstream event, but the downstream ergosterol gene response diverges between the two classes.

We next examined genes related to the echinocandin target and cell wall integrity. *FKS1*, which encodes the catalytic subunit of β-1,3-glucan synthase (the target of echinocandins), was overexpressed in both adaptors. This overexpression may contribute to the tolerance phenotype by increasing the amount of target enzyme.

Among chitin synthase genes, distinct expression patterns were observed between the two adaptor classes. In the Class 3 adaptor, *CHS1* and *CHS2* were down-regulated, while *CHS4* was up-regulated. In the Class 2 adaptor,*CHS1* was down-regulated, whereas *CHS3*, *CHS6*, and *CHS8* were up-regulated. These reciprocal changes in chitin synthase expression suggest that the two adaptor classes may employ different cell wall remodeling strategies to cope with drug exposure.

The *C. auris* genome contains a single chitinase gene, *CHT1*. Notably, *CHT1* was down-regulated in both adaptors, but the degree of suppression was greater in the Class 3 adaptor (5.17-fold) than in the Class 2 adaptor (2.03-fold).

Reduced chitinase activity would be expected to increase cell wall chitin content, a well-documented mechanism of echinocandin tolerance in *Candida* species. The stronger down-regulation of *CHT1* in Class 3 adaptors is consistent with their ability to grow in the presence of caspofungin and micafungin, whereas Class 2 adaptors (which lack echinocandin tolerance) show only modest *CHT1* suppression.

To investigate whether the observed echinocandin tolerance in Class 3 adaptors was associated with cell wall remodeling, we quantified the chitin content of purified cell wall fractions from the same strains used in RNA-Seq, using a colorimetric glucosamine assay following acid hydrolysis.

As shown in supplementary **Supplementary Figure S2**, the parental strain exhibited a basal chitin content of 9.2 ± 0.8% of cell wall dry weight. Class 2 adaptor showed a similar chitin level (9.8 ± 1.5%), which was not significantly different from the parental strain (p = 0.68, unpaired t−test). In striking contrast, Class 3 adaptor displayed a markedly elevated chitin content of 23.7 ± 3.5% (p < 0.001 vs Parent and vs Class 2, one−way ANOVA with Tukey’s post−hoc test), representing an approximate 2.6−fold increase relative to the parental strain.

This substantial chitin accumulation was specifically associated with the echinocandin−tolerant Class 3 phenotype, whereas Class 2 adaptors—which remain fully susceptible to echinocandins—did not exhibit significant cell wall chitin remodeling. These findings suggest that chitin enrichment is a class−defining feature of the echinocandin−tolerant adaptors and may contribute mechanistically to their ability to survive in the presence of caspofungin and micafungin.

Collectively, these transcriptomic data reveal that TCZ−selected adaptors upregulate common resistance determinants (e.g., *MDR1*, *TAC1b*, *UPC2*, *FKS1*), but diverge in ergosterol pathway activation and chitin-related gene expression. The Class 3-specific downregulation of *CHS1*/*CHS2* combined with strong *CHT1* suppression may be particularly important for the echinocandin tolerance phenotype.

## Discussion

In this study, we provide experimental evidence that exposure to the agricultural triazole fungicide TCZ can select for cross-resistance to the clinical azole FLC and, under higher selective pressure, promote tolerance to echinocandins in *C. auris*. Our findings add to the growing body of literature supporting the “One Health” hypothesis that extensive use of azole fungicides in agriculture may serve as an environmental selective force driving the emergence of antifungal resistance in clinically relevant fungal pathogens (Williams et al., 2024; Allison and Hyland, 2025).

In our initial screening of 17 agrochemicals representing diverse chemical classes, only TCZ produced a clear inhibition zone against *C. auris*. The remaining 16 compounds either failed to inhibit fungal growth entirely or exhibited colonies growing within the inhibition zone. This latter phenotype-observed notably for the strobilurins azoxystrobin and pyraclostrobin-suggests that *C. auris* may display tolerance to these compounds under the tested conditions.

Our observation that most non-azole agrochemicals lack significant activity against *C. auris* is consistent with the general understanding that *C. auris* is a human-associated pathogen that has not co-evolved with plant-targeted fungicides. The tolerance phenotype observed for strobilurins, which inhibit mitochondrial respiration by binding to the cytochrome bc1 complex (Bartlett et al., 2002), is notable but not unexpected, as *C. auris* likely possesses intrinsic mechanisms to withstand mitochondrial stressors. Future studies could investigate whether this tolerance involves upregulation of alternative respiratory pathways or efflux−mediated drug extrusion. Nevertheless, our results demonstrate that among the diverse agrochemicals tested, TCZ-a triazole fungicide widely used in postharvest fruit storage-possesses genuine antifungal activity against *C. auris*. This finding prompted us to further investigate whether sub-lethal or lethal exposure to TCZ could select for resistance and cross-resistance to clinical drugs.

We found that brief (48 h) pre-exposure of *C. auris* to 1 μg/mL TCZ (half the MIC) was sufficient to select variants that exhibited reduced susceptibility to both TCZ and the clinical azole FLC, without affecting susceptibility to CSP. The frequency of TCZ−adapted variants was 22/321 (6.9%; 95% CI: 4.5% – 10.1%), which is remarkably high for a short-term exposure, suggesting that *C. auris* possesses a pre−existing genetic or epigenetic heterogeneity that allows rapid adaptation to azole stress. Our findings align with and extend prior work on the emergence of azole cross-resistance following agricultural fungicide exposure. In *Cryptococcus neoformans* and *Cryptococcus gattii*, Bastos et al. demonstrated that TCZ exposure induced *in vitro* cross−resistance to clinical azoles including FLC, itraconazole, and ravuconazole (Bastos et al., 2018). More recently, Peng and colleagues showed that environmentally relevant concentrations of TCZ, difenoconazole, and propiconazole can drive the emergence of triazole-resistant *C. neoformans* in soil through upregulation of *ERG11* and efflux pump genes (Peng et al., 2025a, Peng et al., 2025b). In *A. fumigatus*, the paradigm organism for agricultural-clinical cross-resistance, extensive epidemiological and experimental studies have shown that exposure to agricultural triazoles selects for isolates cross-resistant to clinical azoles. The multi-agency report by European Food Safety et al. (2025) concluded that azole usage outside the human domain is likely or very likely to contribute to the selection of azole-resistant *A. fumigatus* isolates that can cause severe disease in humans (European Food Safety, A et al., 2025). Our work extends this paradigm to *C. auris*, demonstrating for the first time that brief, sub−MIC exposure to TCZ—a concentration that could realistically be encountered on fungicide-treated fruit surfaces—can rapidly select for clinical azole cross-resistance in this critical priority fungal pathogen.

When we exposed *C. auris* to TCZ concentrations above the MIC (4x, 8x, and 16x MIC), we observed the emergence of three distinct phenotypic classes. Most strikingly, the frequency of Class 3 adaptors—which exhibited cross-resistance to TCZ and FLC coupled with tolerance to CSP—increased in a dose-dependent manner as the TCZ selection concentration rose: from 23.0% at 8 μg/mL, to 46.9% at 16 μg/mL, and to 50% at 32 μg/mL. This dose-dependent enrichment strongly suggests that higher TCZ selective pressure actively promotes the emergence of echinocandin tolerance. The distinction between “resistance” and “tolerance” is clinically important. Resistance, evidenced by reduced inhibition zone radius, indicates that the drug’s ability to inhibit the bulk of the fungal population is compromised. Tolerance, defined here as colony growth within an otherwise normal-sized inhibition zone, indicates that a subpopulation of cells can survive drug exposure without altering the drug’s overall efficacy against the majority of the population (Berman and Krysan, 2020; Yang and Berman, 2024). Tolerant cells can serve as a reservoir for the subsequent emergence of full resistance under continued drug pressure, a phenomenon well documented in bacteria and fungi (Balaban et al., 2019; Windels et al., 2019; Verstraete et al., 2022; Arastehfar et al., 2023).

Our observation that Class 3 adaptors are cross-tolerant to both CSP and MCF-as confirmed by disk diffusion and spot assays across a wide concentration range-is particularly noteworthy. This pattern contrasts sharply with the well-characterized CRS−MIS (CSP reduced susceptibility, MCF increased susceptibility) phenotype reported in *C. glabrata* by Healey et al. (2011). In *C. glabrata*, mutants selected for reduced CSP susceptibility paradoxically showed 4- to 32-fold increased susceptibility to MCF, a phenomenon that was shown to be independent of *FKS* mutations and instead involves sphingolipid−mediated alterations in echinocandin−Fks interaction (Healey et al., 2011). In contrast, our *C. auris* Class 3 adaptors displayed tolerance to both echinocandins, with no evidence of differential or paradoxical susceptibility. This distinction strongly suggests that the echinocandin tolerance we observe arises from a fundamentally different mechanism—likely involving cell wall remodeling and chitin accumulation—rather than the sphingolipid-dependent pathway described in *C. glabrata*.

Our RNA-Seq analysis provided molecular insights into the distinct phenotypes of Class 2 and Class 3 adaptors. Both adaptor classes showed overexpression of the multidrug resistance gene *MDR1* and the transcription factor *TAC1b*, consistent with established mechanisms of azole resistance in *C. auris*. Azole resistance in *Candida* species is known to arise from upregulation of efflux pumps (encoded by *MDR1* and *CDR1*) via gain-of-function mutations in transcription factors such as *TAC1* and *MRR1* (ref). Our finding that *CDR1* was overexpressed only in Class 3 adaptors suggests that Class 3 may have acquired additional regulatory changes that further enhance azole efflux capacity. Importantly, only *TAC1b*—not *TAC1a*—was overexpressed in both adaptors, suggesting functional specialization between the two *TAC1* paralogs in *C. auris* (Rybak et al., 2020). The most striking difference between Class 2 and Class 3 adaptors lay in the ergosterol biosynthesis pathway. Class 2 showed broad, coordinated upregulation of nearly all *ERG* genes tested, consistent with a global transcriptional response aimed at increasing ergosterol production to overcome azole-mediated inhibition of Erg11. In contrast, Class 3 exhibited only a restricted upregulation pattern, with approximately half as many *ERG* genes induced. Notably, both classes showed overexpression of *UPC2*, a transcription factor known to regulate ergosterol biosynthesis and sterol uptake. The divergent downstream *ERG* expression patterns suggest that *UPC2* upregulation alone is insufficient to explain the full ergosterol response; additional regulatory inputs likely shape the class−specific expression patterns.

The most informative findings for understanding Class 3−specific echinocandin tolerance came from analysis of cell wall−related genes. *FKS1*, encoding the target of echinocandins, was overexpressed in both adaptors, which may contribute to tolerance by increasing the amount of target enzyme. However, *FKS1* overexpression alone cannot explain the Class 3−specific tolerance, as Class 2 also showed *FKS1* upregulation but remained fully susceptible to CSP. Instead, the key difference appears to involve chitin metabolism. Class 3 adaptors exhibited downregulation of *CHS1* and *CHS2* but upregulation of *CHS4*. More importantly, *CHT1* (the sole chitinase gene in *C. auris*) was downregulated 5.17−fold in Class 3 adaptors, compared to only 2.03−fold in Class 2 adaptors. To validate this transcriptomic prediction, we directly quantified cell wall chitin content. Strikingly, Class 3 adaptors contained 23.7 ± 3.5% chitin (as percentage of cell wall dry weight)—a 2.6−fold increase over both the parental strain (9.2%) and Class 2 adaptors (9.8%, p < 0.001). This chitin accumulation strongly correlates with the degree of *CHT1* suppression, confirming that reduced chitinase activity leads to net chitin deposition. Increased cell wall chitin is a well−documented mechanism of echinocandin tolerance in *Candida* species (Kaneko et al., 2010; Suwunnakorn et al., 2016), and our quantitative data demonstrate that Class 3 adaptors have achieved chitin levels comparable to those previously shown to confer tolerance (Walker et al., 2008, Walker et al., 2013). These findings are consistent with a recent report that *C. auris* adaptation to high caspofungin concentrations correlates with cell wall chitin enrichment (Lara-Aguilar et al., 2021), and extend that observation by identifying suppression of *CHT1*−mediated chitin degradation as the upstream driver of this remodeling.

Taken together, our transcriptomic and biochemical data suggest that TCZ−selected adaptation in C. auris involves: (i) common upregulation of efflux pumps and FKS1; (ii) class−specific activation of the ergosterol pathway; and (iii) Class 3−specific suppression of CHT1 and resultant chitin accumulation as a key mechanism enabling echinocandin tolerance.Our findings have several important implications. First, they provide direct experimental evidence that TCZ-an agricultural triazole widely used on stored fruits-can serve as a selective force for the emergence of clinically relevant antifungal resistance and tolerance in *C. auris*. This aligns with the growing recognition that agricultural fungicide use represents an underappreciated driver of resistance in human fungal pathogens. Second, the dose-dependent enrichment of echinocandin tolerance at higher TCZ concentrations is particularly concerning. Echinocandins are currently recommended as first−line therapy for *C. auris* infections (Centers for Disease Control and Prevention, 2025), and the emergence of tolerance-even in the absence of full resistance-could compromise treatment outcomes. Tolerant cells can persist during drug therapy and may serve as a reservoir for the subsequent evolution of high-level resistance, as has been demonstrated in bacterial systems (Balaban et al., 2019). Our observation that *C. auris* can develop tolerance to both CSP and MCF following agricultural azole exposure suggests that environmental fungicide use could indirectly undermine the efficacy of last-line clinical antifungals. Third, the distinct transcriptomic signatures of Class 2 and Class 3 adaptors suggest that *C. auris* possesses multiple adaptive pathways in response to azole stress. The broader ergosterol pathway activation in Class 2 may represent a “first−line” adaptive response, while the additional chitin-mediated cell wall remodeling in Class 3 may represent a more radical adaptation that also confers echinocandin tolerance. Understanding the genetic basis of these distinct adaptive trajectories-including whether they arise from genetic mutations versus epigenetic changes-represents an important direction for future research.

The concentrations of TCZ used in our experiments warrant consideration in the context of real−world environmental exposures. TCZ is one of the most widely used triazole fungicides globally, applied extensively in both pre−harvest and post−harvest settings to protect fruits, vegetables, and cereals from fungal diseases (Horska et al., 2025). Residue surveillance studies have documented TCZ on a variety of produce: on apples, residues above 0.01 mg/kg persist for more than five months after application, with maximum residue limits established at 1 mg/kg; on pomegranates, terminal whole−fruit residues range from 0.036 to 0.096 mg/kg; and on peaches, residues of 0.24 mg/kg have been reported. In environmental matrices, TCZ exhibits considerable persistence: estimated peak surface water concentrations can reach 13 µg/L for typical agricultural uses, with turf applications generating concentrations of 42–58 µg/L, and soil half−lives ranging from 201 to 433 days in topsoil (Yogendraiah Matadha et al., 2021) (Horska et al., 2025). These data indicate that TCZ residues are not merely transient but can persist in agricultural environments and on produce surfaces for extended periods.

Our sub−MIC exposure concentration (1 µg/mL = 1 mg/L) falls within the range of TCZ concentrations that could realistically accumulate on fruit surfaces, particularly in surface films or post−harvest treatment solutions where fungicide residues become concentrated. While our supra−MIC exposures (8–32 µg/mL) are higher than typical bulk environmental concentrations, they are relevant to scenarios involving direct contact with fungicide-treated surfaces, concentrated residues on produce, or repeated occupational exposures in agricultural settings. Importantly, the dose−dependent enrichment of echinocandin−tolerant variants at higher TCZ concentrations (**Figure 3C**) suggests that the risk of selecting clinically relevant phenotypes increases with the intensity and duration of fungicide exposure.

From a public health perspective, these findings raise several concerns. First, the rapid selection of fluconazole−cross−resistant *C. auris* variants following brief exposure to sub−MIC TCZ (6.9% frequency) suggests that even low−level environmental residues could contribute to the global burden of azole−resistant *C. auris*. This is particularly alarming given that up to 90% of clinical *C. auris* isolates already exhibit fluconazole resistance, and agricultural azole exposure may further constrain therapeutic options. Second, the emergence of echinocandin tolerance—conferring survival in the presence of caspofungin and micafungin without altering the MIC—is clinically relevant because tolerant cells can persist during therapy and potentially serve as a reservoir for the subsequent evolution of high−level resistance (Berman and Krysan, 2020). Echinocandins are currently the recommended first−line therapy for *C. auris* infections, and any factor that compromises their efficacy is of significant clinical concern. Third, the ability of *C. auris* to survive on stored fruit surfaces, combined with the widespread use of TCZ in post−harvest storage, suggests that the food supply chain may serve as an unrecognized route for both exposure and selection of antifungal−resistant C. auris strains.

These observations align with the growing body of evidence supporting the One Health framework, which recognizes that human, animal, and environmental health are interconnected. While direct epidemiological evidence linking agricultural TCZ use to clinical *C. auris* infections remains limited, our experimental data provide proof−of−concept that environmentally relevant TCZ exposure can drive the emergence of clinically significant antifungal phenotypes. We suggest that integrated surveillance spanning agricultural, environmental, and clinical sectors—including monitoring of fungicide residues on produce, screening of environmental *C. auris* isolates for resistance and tolerance phenotypes, and tracking of clinical outcomes—will be essential to fully assess and mitigate the risks posed by agricultural azole use.

The emergence of echinocandin tolerance in TCZ−selected *C. auris* has several clinical implications. First, tolerant cells can persist during therapy, potentially contributing to treatment failure and relapse, as documented in other *Candida* species. Second, tolerance can serve as a stepping−stone to full resistance: prolonged survival under drug pressure provides an extended window for the emergence of high−level resistance mutations—a trajectory already observed in *C. auris* isolates from patients failing echinocandin therapy. Third, standard MIC−based testing cannot detect tolerance, meaning clinicians may unknowingly continue ineffective therapy. Alternative strategies, such as combination with liposomal amphotericin B—to which tolerant strains may show paradoxical susceptibility—warrant investigation, as does the development of accessible tolerance detection methods like diskImageR−based disk diffusion.

Our findings reinforce the need for integrated antifungal stewardship within a One Health framework. We propose four priorities: (i) enhanced surveillance of fungicide residues and environmental *C. auris* isolates for both resistance and tolerance; (ii) regulatory consideration of the cross−resistance and tolerance risks posed by agricultural azoles; (iii) clinical awareness that tolerance is not detected by routine susceptibility testing; and (iv) cross−sector collaboration between agricultural, environmental, and public health agencies to promote judicious fungicide use. Addressing these challenges will require coordinated efforts to mitigate the risk that agricultural practices compromise the efficacy of last−line clinical antifungals.

Several limitations of this study should be acknowledged. First, we performed RNA−Seq on one representative Class 2 adaptor and one representative Class 3 adaptor, comparing each to the parental strain B12037, with three biological replicates per strain. So the generalizability of the transcriptomic signatures across multiple independent adaptors requires confirmation. Second, while our data implicate *CHT1* downregulation and chitin accumulation in echinocandin tolerance, direct measurements of cell wall chitin content and genetic validation (e.g., *CHT1* overexpression or CRISPR−mediated disruption) are needed to establish causality. Third, our experiments were conducted exclusively on a clade III strain (B12037) under defined laboratory conditions; whether other *C. auris* clades—which exhibit substantial genetic and phenotypic diversity, including differences in baseline azole susceptibility and cell wall architecture—show similar adaptive responses to TCZ exposure remains to be determined. Moreover, the selective pressures encountered in environmental or clinical settings (e.g., fluctuating fungicide concentrations, mixed microbial communities, host immune responses) may differ substantially from our experimental conditions and could influence the emergence, stability, and fitness of the phenotypes we observed. Therefore, our findings should be interpreted as proof−of−concept evidence that agricultural triazole exposure can drive clinically relevant antifungal phenotypes in C. auris, rather than as a definitive demonstration of a broad environmental phenomenon. Future studies across multiple clades and under more ecologically relevant conditions will be essential to assess the generalizability and epidemiological significance of these observations. Fourth, we did not perform targeted sequencing of known resistance loci (*ERG11*, *TAC1b*, *UPC2*, and *FKS1* hotspot regions) or whole−genome sequencing of the adapted isolates. Although the resistance and tolerance phenotypes remained stable upon serial passaging in drug−free medium—suggesting a heritable, likely genetic basis—we cannot formally exclude the possibility that epigenetic mechanisms, transcriptional memory, or copy number variations contribute to the observed phenotypes. Future studies employing whole−genome sequencing across multiple independent Class 2 and Class 3 adaptors will be essential to identify the causal mutations driving the class−specific transcriptional programs and to definitively distinguish stable genetic resistance from transient epigenetic adaptation. Future studies should also investigate whether tolerance *in vitro* translates to reduced echinocandin efficacy *in vivo* using animal models of candidiasis. Additionally, screening of clinical *C. auris* isolates for prior agricultural fungicide exposure history-though challenging-could help establish whether the adaptive pathways we identified *in vitro* are relevant to clinical resistance evolution. Fifth, we did not systematically assess fitness costs associated with the adapted phenotypes. Although we qualitatively observed that Class 3 adaptors formed smaller colonies on drug−free medium compared to the parental strain and Class 2 adaptors—suggesting a possible growth disadvantage—we did not quantitatively measure growth rates, doubling times, or competitive fitness under drug−free or drug−containing conditions. Such measurements would be valuable for assessing the stability and epidemiological relevance of the selected variants, as fitness costs may influence the persistence of resistance and tolerance phenotypes in the absence of ongoing selective pressure. Future studies incorporating competitive growth assays and fitness measurements across multiple independent adaptors will be important to determine whether the observed adaptations carry a fitness burden and to predict their likelihood of dissemination in clinical or environmental settings. Sixth, our study tested only a single agricultural triazole (TCZ). TCZ was chosen because it is one of the most widely used triazole fungicides globally and was the only compound among 17 agrochemicals tested that exhibited clear antifungal activity against *C. auris* in our initial screen (**Figure 1**). However, other commonly used demethylation inhibitors (DMIs) in agriculture—such as propiconazole, difenoconazole, and uniconazole—have been shown to select for cross−resistance in other fungal pathogens (Peng et al., 2025a, Peng et al., 2025b; Yu et al., 2025) and may similarly drive adaptive responses in C. auris. Whether these compounds produce phenotypes comparable to those observed with TCZ, or select for distinct adaptive trajectories, remains to be determined. Future studies comparing multiple agricultural triazoles across a broader panel of *C. auris* isolates and clades will be essential to define the generalizability of our findings and to identify structure−activity relationships that may predict the risk of cross−resistance and tolerance selection.

## Conclusions

In summary, this study demonstrates that exposure to the agricultural triazole TCZ can select for *C. auris* variants that are cross-resistant to the clinical azole FLC and, under higher selective pressure, tolerant to the echinocandins CSP and MCF. Transcriptomic analysis reveals distinct adaptive strategies: Class 2 adaptors show broad upregulation of the ergosterol biosynthesis pathway, while Class 3 adaptors exhibit restricted ergosterol activation but suppression of the sole chitinase gene *CHT1*, likely leading to increased cell wall chitin content. Taken together, our findings provide proof−of−concept evidence that exposure to the agricultural triazole TCZ can select for *C. auris* variants that are cross−resistant to the clinical azole FLC and, under higher selective pressure, tolerant to the echinocandins CSP and MCF in a clade III isolate under laboratory conditions. Transcriptomic and biochemical analyses reveal distinct adaptive strategies, with Class 3 adaptors exhibiting *CHT1* suppression and consequent chitin accumulation as a potential mechanism of echinocandin tolerance. While these observations extend the agricultural−clinical cross−resistance paradigm to *C. auris* and highlight the potential for agricultural fungicide use to compromise the efficacy of last−line clinical antifungals, further studies across additional clades and under more environmentally relevant conditions are required to determine the generalizability of these findings.

## Data Availability

The datasets presented in this study can be found in online repositories. The names of the repository/repositories and accession number(s) can be found below: https://www.ebi.ac.uk/arrayexpress/, E-MTAB-16901.
