# Supplementary figures and images for: In vitro exposure to the agricultural triazole tebuconazole selects for fluconazole cross−resistance and echinocandin tolerance in Candidozyma auris
Source: Front Cell Infect Microbiol. 2026 Jul 20;16:1860421. doi: 10.3389/fcimb.2026.1860421 (PMC13429786; doi:10.3389/fcimb.2026.1860421)

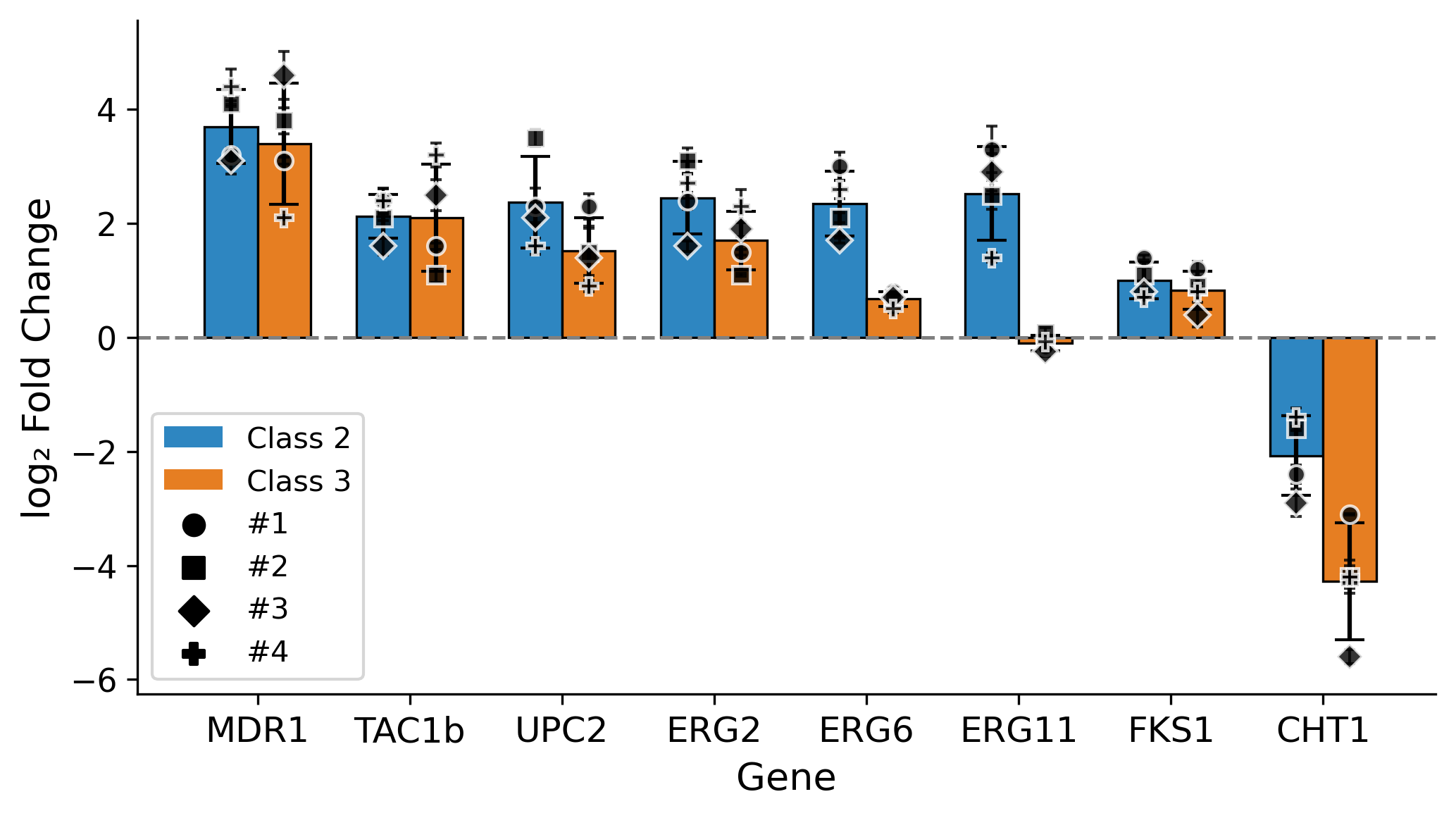

Supplement: Supplementary file 1 [file Image1.tif]

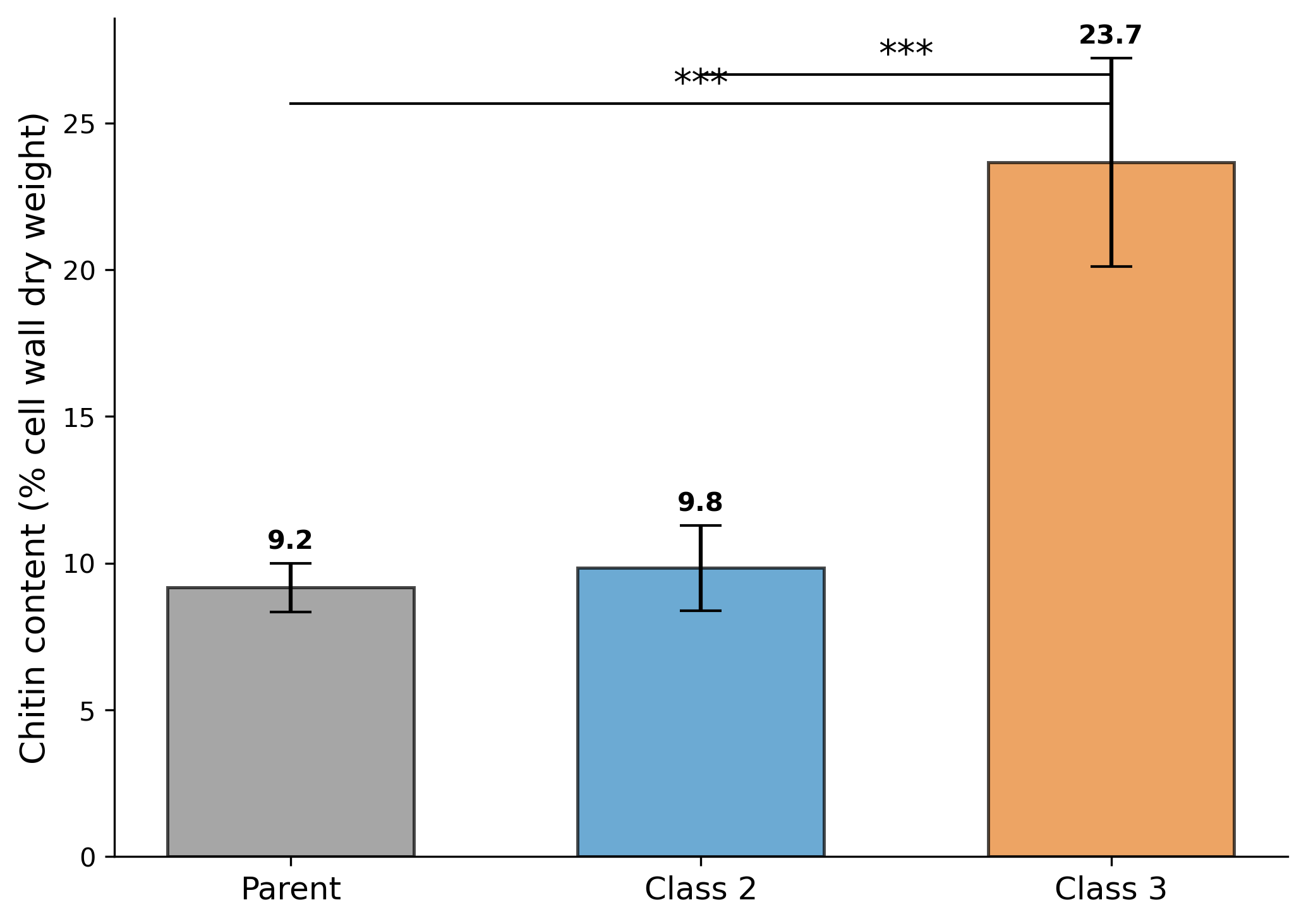

Supplement: Supplementary file 2 [file Image2.tiff]
