# Supplementary material for: In vitro exposure to the agricultural triazole tebuconazole selects for fluconazole cross−resistance and echinocandin tolerance in Candidozyma auris
Source: Front Cell Infect Microbiol. 2026 Jul 20;16:1860421. doi: 10.3389/fcimb.2026.1860421 (PMC13429786; doi:10.3389/fcimb.2026.1860421)
Supplement: Supplementary file 3 [file Table1.docx]

**Supplementary Table S1. Primers used in this study**

| **Primer** | **Sequence** |
| --- | --- |
| ACT1-Fw | TGTCGGTGATGAGGCTCAAT |
| ACT1-Rv | CTCTCTGTTGGACTTGGGGT |
| MDR1-Fw | CTTCTTTGCCAGCCCTTGTT |
| MDR1-Rv | GTCCAGTGGTAGTCAGCCTT |
| TAC1b-Fw | CCCAGTAACAAACCACGCAA |
| TAC1b-Rv | GGATGAATCGGCCAGGTTTC |
| UPC2-Fw | ACGCATAAACCAATCTGCCC |
| UPC2-Rv | CAGAGATACCAGCGCCAGTA |
| FKS1-Fw | GGTGTTGATTTCGCAGGTGT |
| FKS1-Rv | GTGGCCAAAGACTGAGCAAA |
| CHT1-Fw | AACAAGTTCGGTGCTGGTTC |
| CHT1-Rv | TTGAGTCTCGGCCAAGATGT |
| ERG2-Fw | ACATGATCATCTTGCACGCC |
| ERG2-Rv | CTTCACTTGGCCTCTCTCCA |
| ERG6-Fw | TTTGGATGTTGGCTGTGGTG |
| ERG6-Rv | TCACCCTTCACGTACGACAA |
| ERG11-Fw | GGTGCTTATTTTGGTGCCCA |
| ERG11-Rv | TCTCTCTGCACAGCTCGAAA |
