# Supplementary material for: In vitro exposure to the agricultural triazole tebuconazole selects for fluconazole cross−resistance and echinocandin tolerance in Candidozyma auris
Source: Front Cell Infect Microbiol. 2026 Jul 20;16:1860421. doi: 10.3389/fcimb.2026.1860421 (PMC13429786; doi:10.3389/fcimb.2026.1860421)
Supplement: Supplementary file 4 [file Table2.docx]

**Table S2. List of tested agrochemicals with chemical classifications and biological activities**

| **Name** | **Chemical Class** | **Primary Target Crops** | **Target Pathogens /Diseases** | **Mode of action** |
| --- | --- | --- | --- | --- |
| Azoxystrobin | Strobilurin | Cereals, Grapes, Vegetables | Broad-spectrum fungi | Mitochondrial respiration inhibitor (Complex III) |
| Benomyl | Benzimidazole | Fruits, Vegetables, Ornamentals | Powdery mildew, Botrytis | Microtubule assembly disruption |
| Boscalid | Carboxamide | Grapes, Berries, Peanuts | Botrytis, Sclerotinia | Succinate dehydrogenase inhibition |
| Chlorothalonil | Chloronitrile | Potatoes, Tomatoes, Turf | Blights, molds, rusts | Multi-site contact activity |
| Cyprodinil | Anilinopyrimidine | Stone Fruits, Pome Fruits | Botrytis, Scab | Methionine biosynthesis inhibition |
| Dichloran | Aromatic hydrocarbon | Post-harvest (Citrus, Apples) | Post-harvest molds | Membrane disruption |
| Dimethomorph | Morpholine | Grapes, Potatoes, Vegetables | Oomycetes | Cell wall synthesis inhibition |
| Fluopicolide | Benzamide | Potatoes, Tomatoes | Oomycetes | Phospholipid biosynthesis disruption |
| Flutolanil | Carboxamide | Rice, Potatoes, Turf | Basidiomycetes | Succinate dehydrogenase inhibition |
| Iprodione | Dicarboximide | Stone Fruits, Vegetables | Botrytis, Sclerotinia | Osmoregulation disruption |
| Iprobenfos | Organophosphate | Rice | Rice blast | Unknown (anti-blast activity) |
| Isoprothiolane | Dithiolane | Rice | Rice blast | Melanin biosynthesis inhibition |
| Metalaxyl | Phenylamide | Vegetables, Soybeans | Oomycetes | RNA polymerase I inhibition |
| Phthalide | Phthalimide | Rice | Rice blast | Melanin biosynthesis inhibition |
| Pyroquilon | Quinoline | Rice | Rice blast | Melanin biosynthesis inhibition |
| Tebuconazole | Triazole | Wheat, grapes | Powdery mildew, rusts | Ergosterol biosynthesis (C14-demethylase) inhibition |
| Tricyclazole | Triazolobenzothi-azole | Rice | Rice blast | Melanin biosynthesis inhibition |
